# Clinical Phenotyping of Long COVID Patients Evaluated in a Specialized Neuro‐COVID Clinic

**DOI:** 10.1002/acn3.70031

**Published:** 2025-04-08

**Authors:** Luana D. Yamashita, Neel Desai, Abigail R. Manning, Caitlin Pileggi, Sara Manning Peskin, Danielle K. Sandsmark, Dennis L. Kolson, Matthew K. Schindler

**Affiliations:** ^1^ Department of Neurology University of Pennsylvania Philadelphia Pennsylvania USA; ^2^ Department of Biostatistics, Epidemiology, and Informatics University of Pennsylvania Philadelphia Pennsylvania USA

**Keywords:** brain fog, cognitive, long COVID, MoCA, post‐COVID

## Abstract

**Objective:**

To report Long COVID characteristics and longitudinal courses of patients evaluated between 4/14/21–4/14/22 at the University of Pennsylvania Neurological COVID Clinic (PNCC), including clinical symptoms, neurological examination findings, and neurocognitive screening tests from a standardized PNCC neurological evaluation approach.

**Methods:**

This is a retrospective cross‐sectional and longitudinal study in a single‐center tertiary care academic center. Participants include 240 patients with documented evidence of a positive SARS‐CoV‐2 PCR or antibody test who underwent initial evaluation and 182 patients with longitudinal follow‐up. Main outcomes evaluated are patient demographics, duration of illness prior to self‐reported improvement, and cognitive testing results—including the Montreal Cognitive Assessment (version 8.2) (MoCA) and Oral Trail Making Test‐B (OTMT‐B).

**Results:**

The majority (73%) of patients did not require hospitalization for their acute COVID‐19 symptoms. Frequent Long COVID complaints included headache (60%), dizziness/vertigo (40%), and disturbance of taste/smell (40%). Almost all (94%) patients reported cognitive symptoms, and over 30% of patients had abnormal scores on cognitive testing. Severe infection, fewer years of education level, and non‐White race were found to be statistically associated with an increased likelihood of having abnormal scores on cognitive testing. Neuroimaging and clinical laboratory testing were largely not informative for patient care. Sixty‐two percent of patients with follow‐up visits self‐reported improvement in their primary neurological complaint within 1 year of evaluation.

**Interpretation:**

Performance on standardized cognitive screening tests may not be consistent with frequently reported cognitive complaints in Long COVID patients. The most common clinical trajectory was self‐reported improvement in the primary neurological symptom.

## 1 Introduction

Long COVID, also referred to as post‐acute sequelae of COVID‐19 (PASC), consists of heterogenous clinical presentations that result from yet to be defined pathological mechanism(s). Recently, the National Academies of Sciences, Engineering and Medicine (NASEM) Committee on Examining the Working Definition for Long COVID defined Long COVID as “an infection‐associated chronic condition that occurs after SARS‐CoV‐2 infection and is present for at least 3 months as a continuous, relapsing and remitting, or progressive disease state that affects one or more organ systems,” and this definition has been adopted by the Centers for Disease Control and Prevention (CDC) [1]. Conservative estimates of the incidence of Long COVID suggest that ~10% of infected people will experience symptoms, with at least 65 million individuals being affected by Long COVID worldwide [2]. Fatigue is the most commonly reported symptom, but additional symptoms affecting the respiratory, cardiovascular, gastrointestinal, and nervous systems, alone or in combination, are also reported.

Neurological symptoms identified in studies utilizing self‐reported surveys from patients with Long COVID include headache, dizziness and/or vertigo, and cognitive difficulties often described as “brain fog” [3]. Early attempts at characterizing the neurological burden of Long COVID were limited by small sample sizes, reliance on self‐reported symptom measures, retrospective review of data without standardized assessments, and lack of examinations by clinical neurologists [4, 5].

There are few published reports from neurologist‐run Long COVID clinics in the United States. From reports that are available, most neurological testing in this patient population has been within the normal range. When cognitive testing is abnormal, it typically shows impairment in attention and executive function, with relative preservation of memory, visuospatial function, and language [6, 7]. Information on the prognosis and likelihood of recovery of neurological Long COVID is limited due to a lack of data regarding long‐term follow‐up.

Although a few centers have been established to address the needs of patients with neurological symptoms attributed to Long COVID, symptom management varies between institutions, and most clinicians have limited experience with this growing population. The University of Pennsylvania Neurological COVID Clinic (PNCC) was established in April 2021 in response to growing referrals for evaluation of patients with neurological symptoms persisting after an acute COVID‐19 infection. Four clinical neurologists of different subspecialty training developed comprehensive, neurology‐focused historical examinations, and neurological testing. Data from the first year of the PNCC are reported in this study.

## 2 Methods

### 2.1 Study Design

We performed a retrospective analysis of clinical data and testing extracted from the electronic medical record (EMR) system from patients evaluated in the PNCC during its first year of operation (4/14/2021–4/14/2022). The study was conducted in accordance with a University of Pennsylvania Institutional Review Board approved protocol. Patients were included if they demonstrated evidence of a positive SARS‐CoV‐2 PCR or antibody test at least 2 months prior to PNCC evaluation. During this study period, Long COVID diagnosis was defined according to the World Health Organization (WHO) criteria (the continuation or development of new symptoms 3 months after the initial SARS‐CoV‐2 infection, with these symptoms lasting for at least 2 months with no other explanation). Retrospective review of the patient data confirmed that all patients also met the Long COVID NASEM diagnostic criteria.

Demographics, pre‐existing medical and neurological conditions, education level, Body Mass Index (BMI), race, sex assigned at birth, and ethnicity were extracted from the EMR. The patient's acute COVID‐19 infection was categorized as “non‐severe” if managed at home or the emergency department (ED), or “severe” if hospitalized. Neurological complaints were categorized as primary, the single most important complaint to the patient, or secondary, one or more complaints of lesser importance to the patient. Patient complaints were further sub‐divided into categories: cognitive dysfunction, headache, dizziness/vertigo, tinnitus, gait dysfunction, sensory disturbance, visual disturbance, and motor disturbance.

## 3 Procedures

### 3.1 Neurological Examination

A standardized neurological examination and cognitive testing were performed by a board‐certified neurologist as part of the PNCC visit (Supporting Information—S4). The neurological exam included assessment of mental status, cranial nerves, motor system reflexes, sensory system, coordination, and gait. The providers at the PNCC followed the neurologic sequelae initial clinical collecting evaluation recommendations published by the American Academy of Physical Medicine and Rehabilitation (AAPM&R) including obtaining the patient history of present illness, a thorough neurological examination, a review of medication and supplement use, basic lab work dependent on the patient, assessment of previous alcohol use and/or substances, diet/exercise, and changes in activity of daily living [8]. The autonomic nervous system, postural hypotension, and pain were not evaluated in these visits.

Patients were evaluated either in person (*n*: 147) or in a video‐conference telemedicine visit (*n*: 93). Neurological examination was modified for patients seen in video conference visits, as direct evaluation of motor strength, sensation, optic discs, and reflexes could not be evaluated through video evaluation.

### 3.2 Cognitive Assessment

Cognitive testing was performed at the initial evaluation for both in‐person and telemedicine visits. The Montreal Cognitive Assessment (MoCA) version 8.2 was utilized for patients evaluated in the clinic and modified for patients evaluated via telemedicine by omitting visual trails, copy design, and often, the clock drawing. Seventy patients were assessed for clock drawing performance through the telemedicine platform. A loss of more than four points on the MoCA 8.2 was classified as ‘abnormal’ based on published literature [9], with one additional point being granted to patients with less than or equal to 12 years of education. Oral Trail Making Tests A and B (OTMT‐A and OTMT‐B) test scores were adjusted for age‐related norms [10].

### 3.3 Clinical Imaging

If clinical magnetic resonance imaging (MRI) scans were available, these were visually inspected by a neurologist with > 10 years of imaging expertise (MKS) and radiological reports were reviewed. Scans were categorized based on larger lesion burdens than expected based on patient age—visualized as T_2_ hyperintensities, foci of susceptibility, and comparison of age‐expected brain volume.

### 3.4 Laboratory Assessments

Values from laboratory testing ordered as part of clinical care by PNCC providers were extracted. The results for the most commonly ordered blood work included thyroid stimulating hormone, erythrocyte sedimentation rate, vitamin B12, ferritin, and vitamin D. These values were noted as normal or abnormal.

### 3.5 Longitudinal Trajectories of Symptoms

Follow‐up data were available for 181 patients. Primary Long COVID complaints were tracked for notation of change in symptoms (i.e., improving vs. not) based on patient report as documented by a medical provider in a visit. The primary symptom was categorized as improved if the patient reported decreased severity and/or increased manageability of their symptom. The time elapsed between the date of initial infection and the date of subjective improvement was utilized to determine the duration of illness prior to improvement. Treatment administered for the management of symptoms was documented.

### 3.6 Statistical Analysis

Logistic regression was used to assess the association between demographic characteristics and the severity of acute COVID‐19 infection. Likewise, logistic regression was used to assess associations between patient traits and abnormal MoCA scores. Associations between improvement status and patient characteristics were assessed using a Fisher's exact test. Comparisons of MoCA scores between demographically distinct groups were assessed using a Wilcoxon rank—sum test. An association between abnormal MoCA scores and abnormal Oral Trails B Test scores was assessed using a Fisher's exact test. Statistical significance was determined at the (*p* = 0.05) threshold.

## 4 Results

Of 280 patients evaluated in the first year of the PNCC, 240 patients had clinical laboratory testing confirming a positive SARS‐CoV‐2 PCR or antibody test. Patient characteristics separated by the severity of acute infection (“severe” = hospitalization, “non‐severe” = ER or home level care) are reported in Table 1. The population was predominantly female (66%), White (73%), between 40 and 60 years of age (57%) with a median age of 47 years, and highly educated (6% completed college and 26% had at least one additional post‐undergraduate degree). The median time from acute infection to initial PNCC evaluation was 293.5 days (range 42–723 days). Given that most acute infections pre‐dated vaccine availability, only 24 patients had received an initial vaccination, and only 4 patients had received their booster vaccination prior to acquiring COVID‐19. The dates of acute COVID‐19 diagnosis in patients evaluated in the PNCC were consistent with the frequency of acute infections in the Philadelphia region, with more patients infected in the initial COVID‐19 surge in early 2020, followed by a surge due to the Delta variant in late 2020 into early 2021 (Figure S1).

**TABLE 1:** PNCC patient demographics and characteristics.

	Total (*n*: 240) % (*n*)	Non‐severe acute infection (*n*: 176) % (*n*)	Severe acute infection (*n*: 64) % (*n*)
***Age** **(years)***			
18–39	26 (63)	32 (57)	9 (6)
40–59	56 (134)	54 (95)	61 (39)
≥ 60	18 (43)	14 (24)	30 (19)
***Sex***			
Female	66 (159)	69 (121)	59 (38)
Male	34 (81)	31 (55)	41 (26)
***Body Mass Index (BMI)***			
< 30	51 (123)	59 (103)	31 (20)
≥ 30	49 (117)	41 (73)	69 (44)
***Race**/**Ethnicity***			
White	73 (175)	78 (138)	58 (37)
Black	18 (44)	13 (22)	34 (22)
Latino/x	5 (12)	5 (9)	5 (3)
Asian	3 (8)	3 (6)	3 (2)
Other	< 1 (1)	< 1 (1)	0 (0)
***Education***			
High School	11 (27)	7 (12)	23 (15)
Technical/trade	3 (7)	2 (4)	5 (3)
Some College	19 (46)	20 (36)	16 (10)
College Grad	38 (92)	40 (71)	33 (21)
Masters, Doctoral	26 (63)	28 (50)	20 (13)
Unknown	2 (5)	2 (3)	3 (2)
***Pre‐COVID‐19 general diagnoses***	57 (136)	47 (83)	83 (53)
Pulmonary	22 (52)	18 (32)	31 (20)
Cardiac	28 (67)	20 (36)	48 (31)
Endocrine	22 (52)	14 (25)	42 (27)
Renal	1 (3)	0 (0)	5 (3)
Dermatologic	2 (4)	2 (3)	2 (1)
Rheumatologic	9 (22)	7 (13)	14 (9)
Gastrointestinal	8 (19)	9 (16)	5 (3)
Obesity	48 (116)	41 (72)	69 (44)
Other	7 (17)	6 (11)	9 (6)
***Pre‐COVID‐19 neurological diagnoses***	31 (75)	32 (56)	30 (19)
Headache	24 (57)	25 (44)	20 (13)
Epilepsy	< 1 (2)	1 (2)	0 (0)
Neurovascular	2 (4)	< 1 (2)	3 (2)
Neuroinflammatory	< 1 (1)	0 (0)	2 (1)
Neuromuscular	2 (5)	2 (4)	2 (1)
Other	5 (12)	5 (8)	6 (4)
***Pre‐COVID‐19 psychiatric diagnoses***	44 (106)	46 (81)	39 (25)
Anxiety	28 (66)	28 (49)	27 (17)
Depression	30 (72)	32 (57)	23 (15)
Bipolar	5 (11)	5 (9)	3 (2)
PTSD	5 (12)	5 (8)	6 (4)
ADHD	3 (8)	4 (7)	2 (1)

*Note:* Table depicting information on patients grouped by severe and non‐severe acute infection who received care at the PNCC. Demographics include age, sex, body mass index, race, and years of education. Diagnoses prior to COVID‐19 infection are categorized by general, neurological, and psychiatric.

Abbreviations: ADHD, attention‐deficit/hyperactivity disorder; PTSD, post‐traumatic stress disorder.

Age at acute infection and increased BMI were found to be statistically associated (*p* ~0.002) with the severity of acute infection.

At least one pre‐existing neurological diagnosis prior to acute COVID‐19 infection was reported by 31% of patients. Details of the pre‐existing medical and neurological conditions are reported in Table S3. Headache, primarily migraine type, was the most common pre‐existing neurological condition.

### 4.1 Acute COVID‐19 and Long COVID Neurological Symptoms

**FIGURE 1:**
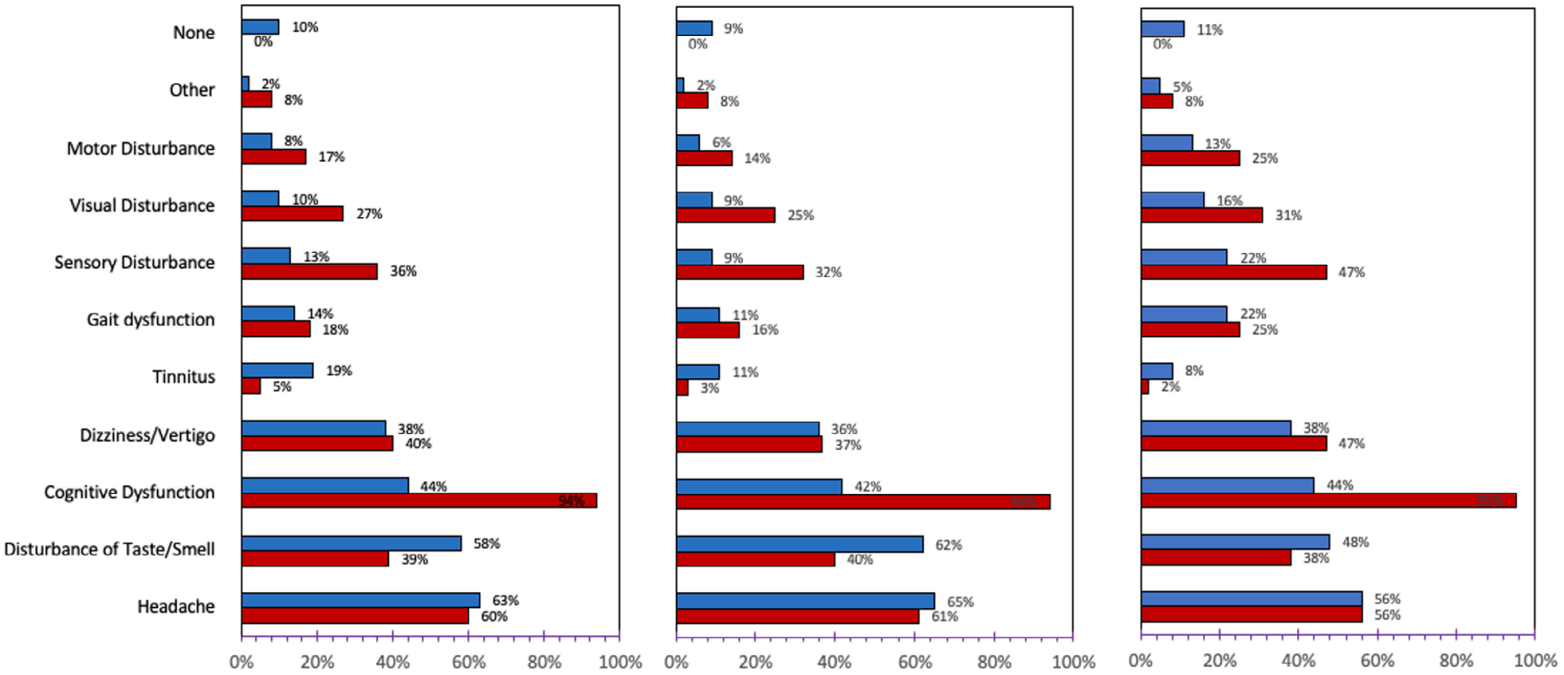
Bar chart depicting the percentage of patients (*x*‐axis) reporting each neurological symptom domain (*y*‐axis) during acute and Long COVID phases of illness. “Other” symptoms refer to pain, tremor, hearing loss, or restless legs syndrome. Left panel refers to the entire cohort (*n*: 240). Middle panel depicts patients with non‐severe infection (i.e., not‐hospitalized or treated at outpatient settings including ED) (*n*: 176). Right panel depicts patients with severe infection (i.e., hospitalization) (*n*: 64). Key: Acute infection (blue), Long COVID (red).

Acute COVID‐19 and Long COVID symptoms reported during the first PNCC evaluation are presented in Figure 1. Ninety‐one percent of patients reported > 3 domains of neurological symptoms, 46% of patients reported > 6 domains of neurological symptoms, and 29% of patients reported > 9 domains of neurological symptoms. Headache and dizziness/vertigo were reported during both acute COVID‐19 infection and Long COVID phases of illness at a similar frequency. Disturbance of taste/smell symptoms was more common in the acute phase, while motor, visual, and sensory disturbances were more often reported in Long COVID. When stratified by illness severity, patients with severe infection experienced more symptoms involving gait, motor, visual, and sensory disturbances during both the acute infection and during Long COVID. Although 44% of all patients experienced cognitive dysfunction during their acute infection, a striking 94% of patients reported cognitive difficulties at their initial Long COVID clinic visit.

Long COVID‐associated cognitive complaints included difficulties with short‐term memory, attention, reading, and multi‐tasking. Patients often referred to these symptoms as “brain fog.” The presence and severity of these symptoms often waxed and waned and, in some people, co‐expressed with increased fatigue. Sixty‐six percent of patients reported cognitive dysfunction as their primary reason for seeking treatment at the PNCC. Following cognitive dysfunction, common primary complaints were headache (14%) and sensory disturbance (9%) (Table S3).

### 4.2 Cognitive Testing

We analyzed three measures of cognitive performance: Abnormal MoCA (> 4 missed), Abnormal OTMT‐B (< 25th percentile), and both Abnormal MoCA and OTMT‐B. Of 218 patients administered the MoCA, 90% (*n*: 197) received OTMT‐B testing. Sixteen percent of patients had abnormal performance for both tests, while 36% of patients were discordant for abnormal testing (i.e., normal on one test, abnormal on the other). When stratified into normal and abnormal scores, a statistically significant association was found between MoCA performance and OTMT‐B using Fisher's exact test (*p* ~0).

**FIGURE 2:**
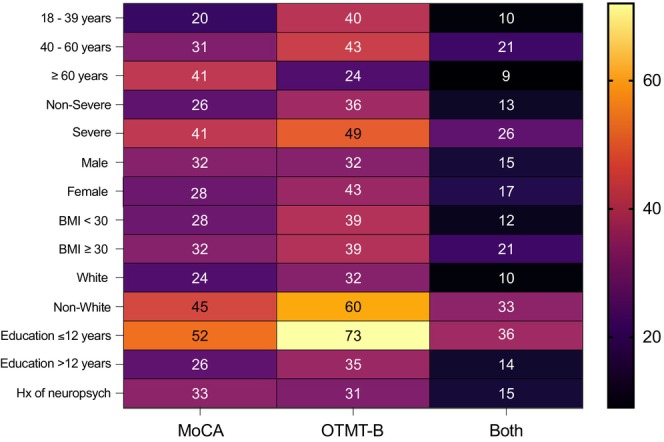
Heat map chart showing the percentage of abnormal testing outcomes by age, severity of acute infection, sex, BMI, race, level of education, and previous history of neuropsychiatric diagnoses. The left panel includes the percentage of patients who missed > 4 points on the MoCA, with a 1 point adjustment for education. The middle panel includes patients who scored < 25th percentile on the OTMT‐B. The right panel includes patients who received abnormal scores in both tests.

Non‐White race, less than 12 years of education, and a history of severe acute infection were associated with abnormal scores in the MoCA and the OTMT‐B. Characteristics of patients with abnormal cognitive testing are displayed in Figure 2 and detailed in Table S3.

Patients with a severe acute infection were more likely to score below the 25th and 9th percentiles on the OTMT‐B. Middle‐aged patients between 40 and 59 years old and young patients 20–39 years old were more likely to score below the 25th percentile compared to older patients 60 years and older, controlling for the severity of acute infection.

Comparisons of MoCA scores between in‐person (*n*: 125) and telemedicine (*n*: 93) patients showed statistically significant associations with a Wilcoxon rank‐sum test (*p* ~0.008). Due to a combination of in‐person and telemedicine evaluations and corresponding total scores, scores were reported in terms of points missed, with missing more than four points being considered abnormal on the MoCA.

Thirty percent of patients administered the MoCA had an abnormal score. Distribution of abnormal scores by category of cognitive test is displayed in Figure S2. Eighty‐one percent of patients missed at least 1 point and 30% missed three or more points in delayed recall. Memory scores improved with category and multiple‐choice cueing, indicating impairment in retrieval rather than encoding. This is a phenotype most associated with an executive pattern of memory impairment. Additional problem areas suggesting impairments in memory/executive function included calculations (41%), chair copy (39%), S‐word generation (25%), abstraction (24%), and clock drawing (24%).

Patients who had a severe acute infection performed worse with a statistically significant association (*p* ~0.0014) in almost all subsections of the MoCA, most notably in forward and reverse digit span, calculations, trails, and chair copy.

Furthermore, statistically significant differences were found for the number of MoCA questions missed between age group (> 45 or ≤ 45) (*p* ~0.0001) and racial group (White vs. non‐White) (*p* ~0.001). Less than 12 years of education was significantly associated with abnormal MoCA scores (*p* ~0.008) even with education adjusted norms. Logistic regression did not show a significant association between duration of illness or prior history of psychiatric diagnoses with abnormal MoCA testing.

### 4.3 Imaging

A brain MRI was available for review in 119 patients, with 59% being classified as normal. Among the abnormal MRI findings were 1 or more chronic microhemorrhages (11%, of which 75% had only one microhemorrhage) and white matter hyperintensities (20% of patients with ≥ 2 hyperintensities). These results were interpreted as chronic nonspecific changes or small vessel ischemic disease and not likely specific to SARS‐CoV‐2 infection. Only 6% of MRIs had abnormal contrast enhancement. One patient's MRI showed enhancement of the olfactory nerves, and another patient's MRI showed enhancement of the facial nerve. These enhancing features were considered to be likely sequelae of SARS‐CoV‐2 infection.

### 4.4 Laboratory Testing

Laboratory testing was not uniformly ordered, but several tests were commonly collected. Six percent (5/96) of patients had an abnormal thyroid‐stimulating hormone test. Erythrocyte sedimentation rate was elevated in 23% (22/94) of patients. Vitamin B12 levels were below range in 41% (35/85). C‐reactive protein was elevated in 23% (14/62), ferritin was elevated in 24% (12/50), and Vitamin D was below range in 25% (20/79) of patients.

### 4.5 Longitudinal Characteristics of Long COVID Symptoms

One hundred and eighty‐two patients had longitudinal follow‐up available for review. Sixty‐two percent of these patients reported improvement in their primary neurological symptoms within 1 year of their initial PNCC visit. Improvement was described as slow, progressive, and gradual over several months. Of those who improved, 43% reported improvement with symptomatic medication directed at treatment for headaches, sensory disturbances, and fatigue. While there are no approved therapies for the cognitive symptoms attributed to Long COVID, patients reported that cognitive therapy was helpful in adapting to limitations. Improvement status was not significantly associated with the patient's age, race, sex, history of psychiatric diagnoses, or MoCA scores (Table 2). Non‐severe infection was found to be significantly associated with a greater likelihood of improvement (*p* = 0.0236), while patients who presented to the PNCC with a longer duration of Long COVID symptoms were associated with a lower likelihood of improving (*p* = 0.049). When grouped by the severity of acute infection, we observed that patients with a cognitive primary complaint improved more slowly than patients with a non‐cognitive primary complaint. On average, patients required about 40–80 days longer to report improvement in cognitive complaints than patients with non‐cognitive complaints (Table 3).

**TABLE 2:** Characteristics of patients with self‐reported improvement in primary complaints.

	Self‐reported improvement % (*n)*	*p*
***Severity of acute infection***		
Severe	48 (22/46)	0.0236
Non‐severe	67 (91/136)	
***Age range***		
18–39	63 (31/49)	0.9586
40–59	62 (64/103)	
≥ 60	60 (18/30)	
***Race group***		
White	62 (83/133)	1.0000
Non‐White	61 (30/49)	
***Sex***		
Male	62 (38/61)	1.0000
Female	62 (75/121)	
***Body Mass Index (BMI)***		
< 30	60 (58/96)	0.7623
≥ 30	63 (54/86)	
***MoCA score***		
Abnormal (missed > 4)	57 (46/81)	0.1497
Normal (missed ≤ 4)	68 (58/85)	
***History of psychiatric diagnoses***		
Present	68 (50/73)	0.1231
Absent	57 (62/109)	

*Note:* Table showing percentage of patients who reported improvement in their primary symptoms within 1 year of initial visit to the PNCC with associated *p*‐values. Characteristics shown include severity of infection, age range, race, sex, BMI, previous history of psychiatric diagnoses, and MoCA score.

**TABLE 3:** Duration of symptoms prior to self‐reported improvement in patients.

Severity of infection (*n*)	Primary symptom % (*n*)	Age (years)	Improved within a year of initial visit % (*n*)	Duration of symptoms prior to self‐reported improvement in patients (days)
Severe acute infection (46)	Cognitive 76 (35/46)	53.0 (60.0–48.5)	46 (16/35)	440 (554.5–275.0) Mean: 444.81
	Non‐cognitive 24 (11/46)	48.0 (54.0–43.5)	55 (6/11)	424.5 (458.5–309.5) Mean: 404.5
Non‐severe acute infection (136)	Cognitive 63 (86/136)	45.5 (57.0–37.25)	67 (58/86)	471 (589.3–329.8) Mean: 487.88
	Non‐cognitive 37 (50/136)	42.0 (49.5–35.0)	66 (33/50)	400 (486.0–288.0) Mean: 404.70

*Note:* Table showing the percentage of patients who reported improvement within a year of their initial visit to the PNCC. Patients are grouped by severity of infection (severe vs. non‐severe), then sub‐grouped by type of primary symptom (cognitive vs. non‐cognitive). Median age and interquartile range of each subgroup are shown. Duration of symptoms prior to improvement in days is shown with median, interquartile range, and mean.

## 5 Discussion

In the PNCC, we collected and analyzed Long COVID neurological symptoms and testing in 240 patients specifically referred for neurological symptoms with laboratory‐confirmed acute SARS‐CoV‐2 infection. Consistent with previous reports, we found that neurological sequelae of Long COVID can manifest following both hospitalized and non‐hospitalized acute COVID‐19 in adults of all ages, races, and health statuses [6, 11]. Cognitive symptoms were nearly ubiquitous, while non‐cognitive complaints, including headache, sensory disturbance, and dizziness/vertigo, were also common. Neuroimaging was considered normal, age appropriate for most patients, indicating no gross structural injury secondary to SARS‐CoV‐2 infection. The infrequent abnormal findings were considered to be unrelated to SARS‐CoV‐2 infection or Long COVID symptoms. Nearly two‐thirds of patients who underwent a follow‐up evaluation reported improvement in their primary neurological complaint, a median of 12 months following their acute infection.

Memory, executive/visual spatial function, and language were most likely to be impaired on MoCA testing results. Our findings are consistent with prior studies reporting impairments in delayed recall in patients with Long COVID [7, 11]. Interestingly, although only 10% of patients missed one point on the MoCA Alternating Trail Making test, 39% of patients scored below the 25th percentile of the age‐adjusted normative values on the OTMT‐B. This discrepancy may be attributed to a shorter trails test on the MoCA, the aid of visual cues compared to the OTMT‐B, or age‐adjusted normative values for OTMT‐B [10]. Despite 94% of patients expressing cognitive difficulties, validated cognitive assessments (MoCA and OTMT‐B) were abnormal in only 30% and 39% of patients respectively, with only 16% of patients having abnormal scores for both tests. The relatively low percentage of abnormal cognitive assessment scores in comparison to reported cognitive difficulties in our cohort suggests the MoCA and OTMT‐B may not be sensitive for detecting the cognitive impairments experienced by Long COVID patients. Our results support a previous study of Long COVID patients that demonstrated the MoCA was only 50% sensitive and 63.3% accurate at detecting low/extremely low performance versus normal performance [12]. These limitations should be considered when evaluating cognitive impairment in this population.

Persistent neurological symptoms following a viral infection including Epstein–Barr virus (EBV), Influenza, Ross River virus, and other conditions with similar qualitative symptomatology such as myalgic encephalomyelitis/chronic fatigue syndrome (ME/CFS) have been reported prior to COVID‐19. Long COVID shares many features with these conditions including neurocognitive difficulties, mood disturbance, and fatigue persisting for 6 months or longer [13, 14]. Similar to the patients in our cohort, patients recovering from neuro‐invasive West Nile virus (WNV) demonstrate mild impairments in immediate and delayed recall [15], and patients with previous chikungunya infection have worse performance in MoCA compared to healthy controls [16]. In studies comparing Long COVID to ME/CFS, both groups had impaired processing speed, sustained attention, and verbal memory, but ME/CFS patients performed worse in attention and visual perception with longer lasting symptoms [17]. More research with longer follow‐up periods is needed to better compare Long COVID trajectories to other conditions.

Sixty‐two percent of our patients with longitudinal follow‐up reported subjective improvement of their primary neurological symptom within a median of 12 months after acute COVID‐19. Subjective measures of improvement indicated the patients reported decreased severity, which may have reflected clinical improvement or better manageability of their primary Long COVID symptom. Some patients may have experienced secondary neurological Long COVID complaints or non‐neurological complaints, which were not assessed for improvement. Improvement in most of our cohort aligns with converging evidence to suggest that a characteristic of Long COVID is significant improvement in most people, as recently indicated by the CDC [18]. Other studies have reported prolonged symptom duration and slow improvement [19, 20]. Symptom improvement in our cohort remained consistent across sex, age, and race, but patients who had more severe acute COVID‐19 infections were less likely to report improvement. This result is consistent with a systematic analysis that showed patients admitted for severe infection took almost twice as long to improve Long COVID symptoms compared to those who had mild acute infection [21]. Improvement was also less common in patients with a longer duration of symptoms. However, this observation may have been influenced by self‐selection bias, as patients who are not improving or are taking longer to improve are more likely to have been referred to the clinic and are more likely to have attended follow‐up visits. Many of our patients participated in cognitive and/or physical therapy and received symptomatic treatment, but the lack of standardized therapies/treatments available to many of the patients evaluated in the PNCC limits the ability to interpret the role of these factors on clinical courses. Understanding differences in patient characteristics between those who experience varying degrees of improvement versus patients who experience complete symptom resolution is still important and under‐studied. Further research with longer follow‐up periods and more standardized measures is necessary to differentiate between subjective improvement of symptoms, as reported in our study, and improvement of functional status, which includes the ability to work and perform usual activities.

There are few neurologist‐run clinics dedicated to Long COVID, and the prevalence of neurological symptoms far outpaces their capacity. It is challenging for neurologists to diagnose Long COVID when working definitions remain broad with long timeframes from infection to symptom development and many symptoms overlap with other neurological and systemic diseases. The NASEM definition states that “Long COVID can exacerbate pre‐existing health conditions or present as new condition,” making any exacerbation of a previously occurring neurological symptom (e.g., migraine headache, sleep disturbance, or cognitive impairment) a potential manifestation. Given the wide prevalence of acute COVID‐19 infection, broad characterization of Long COVID symptoms, and an onset timeframe up to 1 year after an acute infection per NASEM definition, there is a potential risk for patients being misdiagnosed with Long COVID. Without standardized testing and disease‐specific criteria, a Long COVID diagnosis should be approached carefully‐ only if other etiologies have been ruled out and pre‐existing disorders are identified as stable or altered. Notably, the NASEM definition of Long COVID will almost certainly be modified (within 3 years) as more data are accumulated [1]. As the number of patients seeking care for Long COVID grows, we believe that general neurological clinics are as well‐equipped as specialized clinics to provide structured neurological assessments, symptom‐based treatments, and referrals to physical/cognitive therapy. Many patients with Long COVID undergo extensive testing, which can be expensive and time consuming. In our experience, routine testing including brain MRI and routine clinical laboratory testing did not add to clinical management and treatment. As most of the patients reported slow improvement after an acute infection, limiting testing to patients who do not follow this trajectory may be a more judicious use of resources. Given the systemic symptoms that characterize the heterogeneity of Long COVID, a multidisciplinary approach could be more helpful for consolidated triage/treatment than specialized neurological care for this specific patient population [22].

Our study features notable strengths, including a large sample size of 240 patients, and consistency across standardized clinical interviews, neurological examinations, and patient monitoring. Important limitations include subjective measures of improvement based on patient reports within the context of a real‐world clinical setting, not clinical research trials. Additional standardized testing and functional measures, including abilities to perform usual activities, were not captured. The autonomic nervous system, postural hypotension, and pain were not evaluated. Additionally, the single center design recruiting primarily from one geographic region limits the generalizability of our findings. We cannot reach conclusions about the prevalence of neurological symptoms of Long COVID in the general population as our patients were specifically referred for evaluation of neurological symptoms. Our patient population was primarily White, female, and highly educated, which may reflect this group's ability to seek and obtain healthcare, rather than a higher occurrence of Long COVID symptoms in this cohort. Follow‐up data was not available for some of the patients, which may have skewed the reported percentage of improvement, because people who had improved may have been less likely to present for a follow‐up. Most patients included in the study had not received a vaccination, so our study cannot inform on the effects of the vaccine on Long COVID symptoms. Finally, our data informs on variants of SARS‐Cov‐2 in 2021, which may not be applicable to current circulating variants.

## Author Contributions

Conceptualization, Luana D. Yamashita, Abigail R. Manning, and Matthew K. Schindler; methodology, Luana D. Yamashita, Abigail R. Manning, Caitlin Pileggi, Sara Manning Peskin, Danielle K. Sandsmark, Dennis L. Kolson, and Matthew K. Schindler; formal analysis, Luana D. Yamashita and Neel Desai; data curation, Luana D. Yamashita, Abigail R. Manning, Neel Desai, and Matthew K. Schindler; writing – original draft preparation, Luana D. Yamashita, Neel Desai, and Matthew K. Schindler; writing – review and editing, Luana D. Yamashita, Neel Desai, Abigail R. Manning, Caitlin Pileggi, Sara Manning Peskin, Danielle K. Sandsmark, Dennis L. Kolson, and Matthew K. Schindler; supervision, Matthew K. Schindler; All authors have read and agreed to the published version of the manuscript.

## Conflicts of Interest

The authors declare no conflicts of interest.

## Supporting information


**Figure S1.** Bar graph depicting number of patients (*y*‐axis) affected by variant type corresponding to month and year (*x*‐axis). Variants were extracted from SARS‐CoV‐2 Variants Circulating in the Delaware River Valley Tracked by Surveillance Sequencing (https://microb120.med.upenn.edu/data/SARS‐CoV‐2/). B.1 (blue), Other (red), Alpha (green), Delta (purple), Omicron (orange).

**Figure S2.** Bar graph depicting percentage of patients (*x*‐axis) who received abnormal scores on sections of the MoCA (*y*‐axis). Patients are grouped by severity of infection. “Non‐severe” patients (*n*: 160) (red), “Severe” patients (*n*: 58) (pink) and “Total” patients (*n*: 218) (blue).

**Table S1.** Supporting Information.

**Data S1.** Supporting Information.

## Data Availability

The data that support the findings of this study are available on request from the corresponding author. The data are not publicly available due to privacy or ethical restrictions.
