# Corrigendum to “Clinical Phenotyping of Long COVID Patients Evaluated in a Specialized Neuro‐COVID Clinic”

**DOI:** 10.1002/acn3.70069

**Published:** 2025-05-19

Yamashita, L.D., Desai, N., Manning, A.R., Pileggi, C., Peskin, S.M., Sandsmark, D.K., Kolson, D.L. and Schindler, M.K. (2025), Clinical Phenotyping of Long COVID Patients Evaluated in a Specialized Neuro‐COVID Clinic. *Annals of Clinical and Translational Neurology*, https://doi.org/10.1002/acn3.70031

In Paragraph 1 of Section 4 “Results” section, the text “The population was… highly educated (6% completed college and 26% had at least one additional post‐undergraduate degree)” was incorrect. This should have read: “The population was… highly educated (65% completed college and 26% had at least one additional post‐undergraduate degree).”

We apologize for this error.

