# Supplementary figures and images for: Clinical Phenotyping of Long COVID Patients Evaluated in a Specialized Neuro‐COVID Clinic
Source: Ann Clin Transl Neurol. 2025 Apr 8;12(6):1126–34. doi: 10.1002/acn3.70031 (PMC12172097; doi:10.1002/acn3.70031)

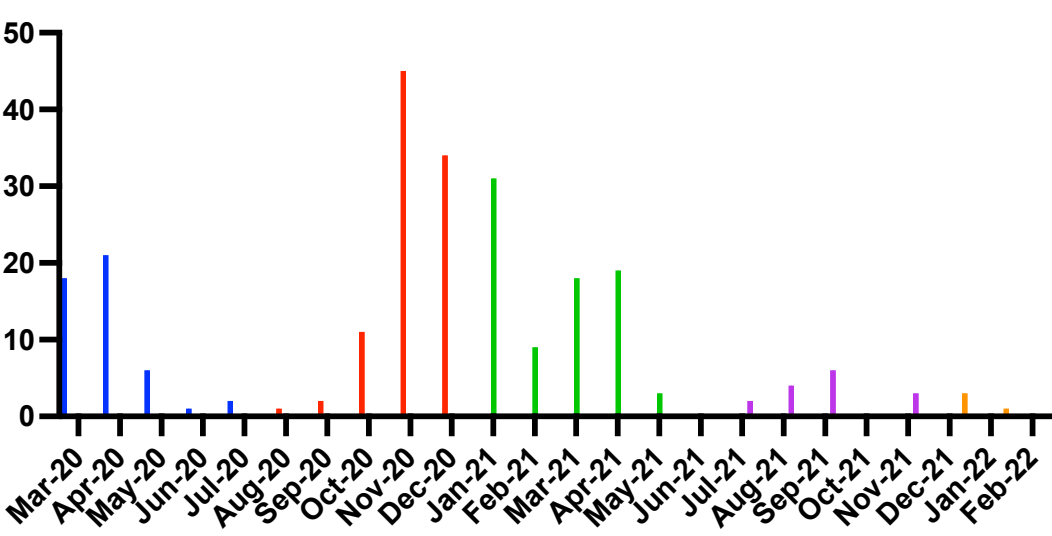

Supplement: Supplementary file 1 — Figure S1. Bar graph depicting number of patients (y‐axis) affected by variant type corresponding to month and year (x‐axis). Variants were extracted from SARS‐CoV‐2 Variants Circulating in the Delaware River Valley Tracked by Surveillance Sequencing (https://microb120.med.upenn.edu/data/SARS‐CoV‐2/). B.1 (blue), Other (red), Alpha (green), Delta (purple), Omicron (orange). [file ACN3-12-1126-s001.pdf]

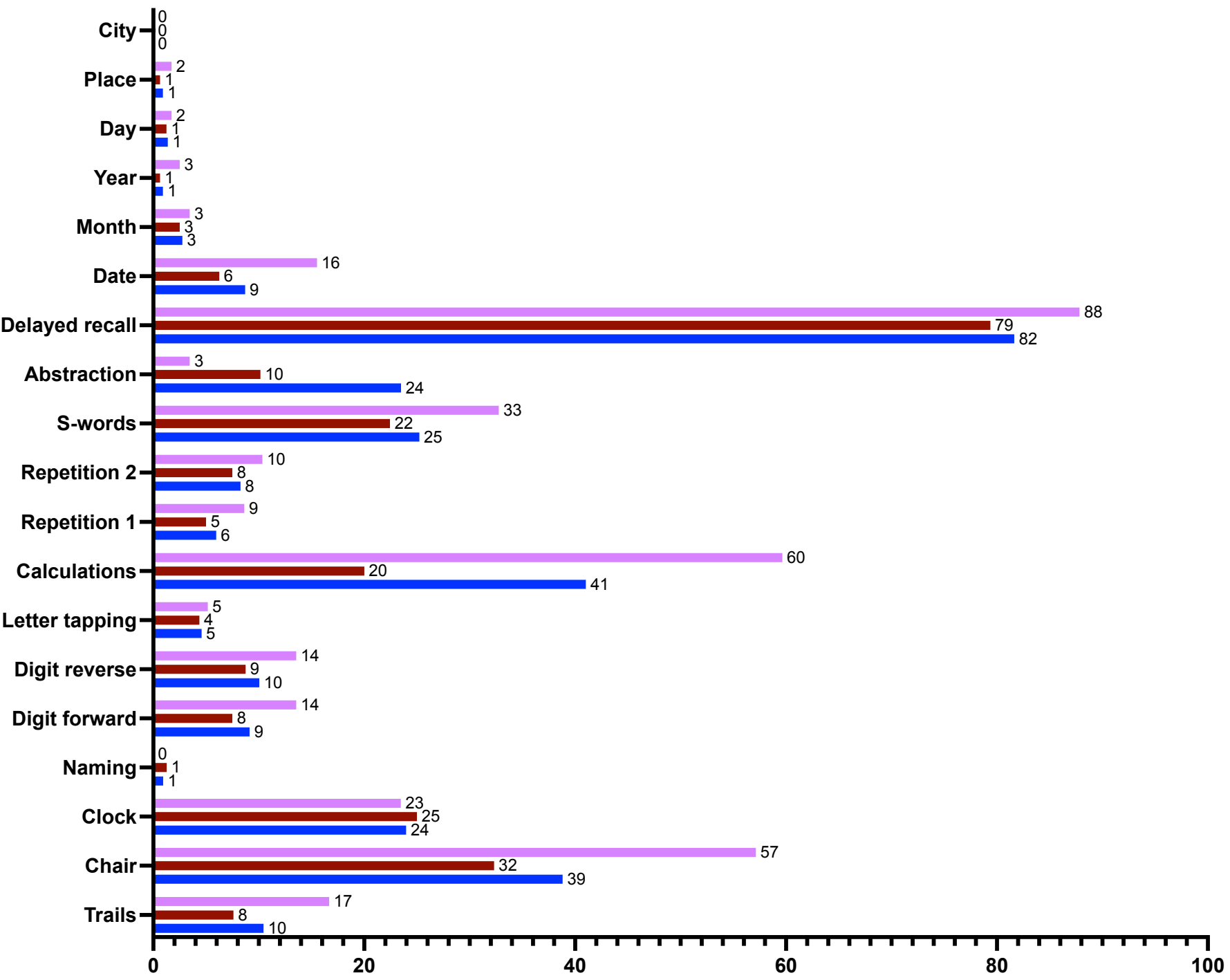

Supplement: Supplementary file 2 — Figure S2. Bar graph depicting percentage of patients (x‐axis) who received abnormal scores on sections of the MoCA (y‐axis). Patients are grouped by severity of infection. “Non‐severe” patients (n: 160) (red), “Severe” patients (n: 58) (pink) and “Total” patients (n: 218) (blue). [file ACN3-12-1126-s002.pdf]
