# Supplementary material for: Clinical Phenotyping of Long COVID Patients Evaluated in a Specialized Neuro‐COVID Clinic
Source: Ann Clin Transl Neurol. 2025 Apr 8;12(6):1126–34. doi: 10.1002/acn3.70031 (PMC12172097; doi:10.1002/acn3.70031)
Supplement: Supplementary file 3 — Table S1. Supporting Information. [file ACN3-12-1126-s003.docx]

**Supplemental Table 1:** Pre-existing diagnoses prior to COVID-19 infection

| Pre-COVID-19 Medical Diagnosis (n:136) | 57%  (136/240) | Pulmonary: (n:52)  Cardiac (n:67)  Endocrine (n:52)  Renal (n:3)  Dermatologic (n:4)  Rheumatologic (n:22)  GI (n:19)  Obesity (n:116)  Other (n:17) | Asthma (n:30), OSA (n:20)  Hypertension (n:49), Arrhythmia (n:7), DM2 (n:28), Hypothyroidism (n:25)  CKD (n:3)  Psoriasis (n:3)  RA (n:6), SLE (n:3), Sarcoid (n:2)  IBS (n:8), UC (n:2), Crohn’s (n:2)  Morbid obesity (n:33)  Cancer (n:7), HIV (n:3) |
| --- | --- | --- | --- |
| Pre-COVID-19 Neurological Diagnosis (n:75) | 31%  (75/240) | Headache (n:57)  Epilepsy (n:2)  Neurovascular (n:4)  Neuroinflammatory (n:1)  Neuromuscular (n:5)  Other (n:12) | Migraine (n:42), Tension headache (n:15), Stroke (n:2)  TIA (n:1), SAH (n: 1),  Possible Neurosarcoidosis (n:1), Neuropathy (n:2), Brachial plexus injury (n:1), Muscle weakness (n:2), sleep disorders (n:2) |

**Sup Table 1:** Table showing the breakdown of medical and neurological diagnoses reported by patients. Abbreviations: OSA = Obstructive sleep apnea, DM2 = Diabetes Mellitus Type 2, CKD = Chronic kidney disease, RA = Rheumatoid arthritis, SLE = Systemic lupus erythematosus, IBS = Irritable bowel syndrome, UC = Ulcerative colitis, HIV = Human immunodeficiency virus, TIA = Transient ischemic attack, SAH = subarachnoid hemorrhage

**Supplementary Table 2:** Primary and Secondary Long COVID neurological complaints

| **Neurological symptom** | **Primary Long COVID complaint (n: 240)**  **% (n)** | **Secondary Long COVID complaint (n:240)**  **% (n)** |
| --- | --- | --- |
| Cognitive dysfunction | 66% (158) | 94% (226) |
| Headache | 14% (33) | 60% (143) |
| Sensory disturbance | 9% (22) | 36% (87) |
| Other | 8% (20) | 8% (19) |
| Dizziness/vertigo | 2% (4) | 40% (94) |
| Visual disturbance | <1% (1) | 27% (64) |
| Tinnitus | <1% (2) | 5% (13) |
| Gait dysfunction | 0% (0) | 18% (44) |
| Disturbance of Taste/Smell | 0% (0) | 40% (94) |
| Motor disturbance | 0% (0) | 17% (41) |

**Supp Table 2**: Neurological symptoms reported as primary and secondary Long COVID complaints by patients. ‘Primary’ referred to the single most important complaint affecting the patient. ‘Secondary’ referred to one or more complains that were less impactful to the patient. Other’ symptoms refer to pain, psych symptoms, fatigue, tremor, hearing loss, and restless-leg syndrome.

**Supplementary** **Table 3:** Characteristics of patients with abnormal testing outcomes

|  | MoCA (>4 missed)  % (n) | OTMT-B  (<25^th^ percentile)  % (n) | Both Abnormal MoCA and OTMT-B  % (n) |
| --- | --- | --- | --- |
| Total | 30% (65/218) | 39% (79/201) | 16% (32/197) |
| Age  18-39 years  40-59 years  60+ years | 53 (25-76)  20% (11/56)  31% (38/123)  41% (16/39) | 47 (25-74)  40% (21/53)  43% (50/115)  24% (8/33) | 49 (25-74)  10% (5/52)  21% (24/112)  9% (3/33) |
| Severity  Non-hospitalized    Hospitalized | 26% (41/160)  41% (24/58) | 36% (54/150)     49% (25/51) | 13% (19/147)  26% (13/50) |
| Sex  Female  Male | 28% (41/144)  32% (24/74) | 43% (58/135)    32% (21/66) | 17% (22/132)  15% (10/65) |
| BMI  < 30           > 30 | 28% (31/111)  32% (34/107) | 39% (40/102)  39% (39/99) | 12% (12/100)  21% (20/97) |
| Race  White    Non-white | 24% (39/160)  45% (26/58) | 32% (47/148)    60% (32/53) | 10% (15/145)  33% (17/52) |

**Supplementary** **Table 3 (Continued):** Characteristics of patients with abnormal testing outcomes

| Duration of illness (in days, median, IQR) | 273 (63-707) | 291 (42-716) | 273 (100-707) |
| --- | --- | --- | --- |
| Education  $\leq$ 12 years  >12 years  Unknown | 52% (13/25)  26% (50/190)  67% (2/3) | 73% (16/22)  35% (63/178)  0% (0/1) | 36% (8/22)  14% (24/174)  0% (0/1) |
| History of psychiatric diagnoses | 33% (28/85) | 31% (23/75) | 15% (11/75) |

**Sup Table 3:** Table depicting characteristics of patients who received abnormal cognitive test scores. Demographics include age, severity of infection, sex, BMI, race, education and history of psychiatric diagnoses. Duration of illness is time elapsed from acute-infection and initial visit at the PNCC. Left panel includes percentage of patients who missed >4 points on the MoCA, with 1 point adjustment for education. Middle panel includes patients who scored <25^th^ percentile on the OTMT-B. Right panel includes patients who received abnormal scores in both tests.
