# Supplementary material for: Clinical Phenotyping of Long COVID Patients Evaluated in a Specialized Neuro‐COVID Clinic
Source: Ann Clin Transl Neurol. 2025 Apr 8;12(6):1126–34. doi: 10.1002/acn3.70031 (PMC12172097; doi:10.1002/acn3.70031)
Supplement: Supplementary file 4 — Data S1. Supporting Information. [file ACN3-12-1126-s004.docx]

**History of Present Illness:**

| **PreCOVID History** | | |
| --- | --- | --- |
| **Pre-COVID Neuro/Psych History:** | | |
| Headache/Migraine: |  | |
| Stroke/TIA: |  | |
| MS/Neuroinflammatory: |  | |
| Seizure/epilepsy: |  | |
| Neuromuscular: |  | |
| Psychiatric: |  | |
| Neuropathy: |  | |
| Movement : |  | |
| Other: |  | |
| **Pre-COVID Medical History:** | | |
| Pulm: |  | |
| Cardiac: |  | |
| Diabetes/endocrine: |  | |
| Renal: |  | |
| Rheum: |  | |
| GI: |  | |
| Derm: |  | |
| TBI: |  | |
| Other: |  | |
| **Substance abuse history:** | | |
| Smoking: |  | |
| ETOH: |  | |
| Other: |  | |
| **Education:** | | |
| Highest level: | |  |
| **Work:** | | |
| Pre-covid: |  | |
| Post-covid (document change in performance, any accommodations, etc: |  | |
| **Handedness:** |  | |
| **Family History (neuro):** |  | |
| **COVID Vaccination History:** | | |
| Date(s) of initial vaccine series: |  | |
| Manufacturer: |  | |
| Dates of boosters |  | |
| Manufacture(s): |  | |

| **Acute COVID Infection History** | |
| --- | --- |
| COVID symptom onset:date: |  |
| COVID PCR test date: |  |
| COVID antibody test date: |  |
| **Level of care**  (Home, ER (# times), inpatient, ICU): |  |
| Dates of admission/discharge  # ER/inpatient visits: |  |
| **Acute COVID Symptoms (non-neuro)**  (Asymptomatic, SOB, fever, cough, chills, myalgias, palpitations, GI, general fatigue, other): |  |
| **Acute COVID Symptoms (neuro)**  (None, headache, paresthesias, focal weakness), dizziness, vertigo, gait dysfunction, visual disturbance, loss of smell, loss of taste, tinnitus, cognitive dysfunction, other): |  |
| **Acute COVID-associated Neurological event**  (GBS, seizure, stroke/TIA, encephalitis, meningitis, Bell's palsy, other): |  |
| **COVID Treatments**  (Supportive (no O2), supportive (O2), antivirals, steroids, plasma/IVIG, monoclonal antibodies, other): |  |
| **Detailed HPI:** |  |

| **Post-COVID - Chronic Neurologic Sequalae:** | |
| --- | --- |
| **Ongoing Symptoms (COVID neuro review of systems) - comment on new or worsening of preexisting:** | |
| Vision: |  |
| Sensory: |  |
| Motor: |  |
| Ambulation: |  |
| Coordination: |  |
| Energy: |  |
| Cognition: |  |
| Sleep: |  |
| Mood: |  |
| Headache: |  |
| Dizziness: |  |
| Smell: |  |
| Taste: |  |
| Other: |  |
| **Symptomatic Medications for neurological symptoms:** |  |
| **Non-Pharmacologic Treatment (Ordered prior to your evaluation)** | |
| PT/OT: |  |
| Cognitive therapy: |  |
| Psychotherapy: |  |
| Other: |  |
| Other Specialists Seen/referred: |  |

| Primary post-COVID Neurological complaint **choose one: headache, cognitive, dizzy/vertigo, sensory, motor, other** | Other: |
| --- | --- |
| Secondary post-COVID Neurological complaint(s): **headache, cognitive, dizzy/vertigo, sensory, motor, other** | Other: |

Current Outpatient Medications:

**Neurologic Examination:**

General

Cognitive:

| Formal Cognitive Testing:  MOCA version 8.2: | |
| --- | --- |
| Trails |  |
| Chair copy: |  |
| Clock Draw |  |
| Naming |  |
| Encoding trial 1: |  |
| Encoding trial 2: |  |
| Digits forward: |  |
| Digits reverse: |  |
| Letter tapping: |  |
| Calculations |  |
| Repetition 1 (robber): |  |
| Repetition 2 (student): |  |
| S-word score > 11: |  |
| Total s-words: |  |
| Repetitions: |  |
| Abstraction: |  |
| Delayed recall Total: |  |
| W/category cues: |  |
| W/ multiple choice: |  |
| Orientation date: |  |
| Orientation month: |  |
| Orientation year: |  |
| Orientation day: |  |
| Orientation place: |  |
| Orientation city: |  |
| Total score (30): |  |
| **Oral trails A:** | |
| Time to 25 (sec): |  |
| **Oral trails B::** | |
| Time to 13 (sec)  1-A, 2-B, 3-C, 4-D, 5-E, 6-F, 7-G, 8-H, 9-I, 10-J, 11-K, 12-L, 13: |  |
| **Forward digit span (optional:** | |
| Highest achieved  1-8-4 / 2-7-9  4-1-6-2 / 8-1-9-5  6-4-9-2-8 / 7-3-8-6-1  3-9-2-4-7-5 / 6-2-8-3-1-9  9-6-4-7-1-5-3 / 7-4-9-2-6-8-1  4-7-2-5-8-1-3-9 / 2-9-5-7-3-6-1-8  6-8-4-1-9-3-5-2-7 / 1-3-9-2-7-5-8-6-4: |  |
| **Reverse digit span (optional):** | |
| Highest achieved  2-5 / 4-7  2-9-6 / 3-7-4  7-1-8-6 / 5-1-6-3  5-2-4-9-1 / 9-1-7-3-6  6-8-5-7-9-2 / 8-1-6-3-5-9  1-5-2-9-7-3-8 / 7-3-1-6-8-5-2  3-6-4-9-5-2-7-1 / 6-3-5-7-1-8-2-9: |  |

Cranial Nerves

| *II* | VFF, PERRL, no APD, sharp disc margins and no pallor. |
| --- | --- |
| *III, IV, VI* | Full extraocular movements, no nystagmus, no ptosis, pursuits were smooth |
| *V* | Symmetric facial sensation. |
| *VII* | Symmetric facial movements and nasolabial folds. |
| *VIII* | Hearing intact bilaterally. |
| *IX, X* | Uvula midline, elevates symmetrically with phonation. |
| *XI* | 5/5 strength of SCM and trapezius. |
| *XII* | Midline tongue protrusion, no fasciculations. |

Motor

Arms and legs had normal bulk, tone and were strong (5/5) to confrontational testing, except if noted below.

| Abnormal motor (R): |  |
| --- | --- |
| Abnormal motor (L): |  |

Reflexes

Reflex testing of the bilateral arms and legs were 2+ and symmetric, plantar responses were flexor, except if noted below

| Abnormal reflexes (R): |  |
| --- | --- |
| Abnormal reflexes (L): |  |

Sensory

Sensation to light touch, temperature/pain, vibration/proprioception were intact and symmetric except if noted below,

| Abnormal sensation (R): |  |
| --- | --- |
| Abnormal sensation (L): |  |
| Romberg: |  |

Coordination

Coordination was normal including finger-to-nose testing and rapid alternating movements except if noted below.

| Abnormal coordination (R): |  |
| --- | --- |
| Abnormal coordination (L): |  |

Gait

Gait was narrow-base, normal speed, not ataxic, and not spastic except if noted below

| Abnormal gait: |  |
| --- | --- |

**Data:** Independent visualization of images was performed (not review of report) unless otherwise specified.

| **Data** |  |
| --- | --- |
| **Most recent Brain MRI -Date/protocol (penn/osh,TBI):** |  |
| Abnormal brain MRI findings: |  |
| **Most recent C-spine MRI - Date:** |  |
| Abnormal c-spine MRI findings: |  |
| **Most recent T-spine MRI -Date:** |  |
| Abnormal t-spine MRI findings: |  |
| **LP - Date:** |  |
| LP findings: |  |
| **EMG/NCS -Date:** |  |
| EMG/NCS findings: |  |
| Labs: |  |

**Assessment and Plan:**
